# Fungal Infections in Severe Acute Pancreatitis: Insights from a Case Series

**DOI:** 10.3390/jcm15020790

**Published:** 2026-01-19

**Authors:** Andreea Iacob, Gheorghe G. Balan, Mihaela Blaj, Adi-Ionut Ciumanghel, Vasile Sandru, Elena Toader

**Affiliations:** 1 Faculty of Medicine, Grigore T. Popa University of Medicine and Pharmacy, 700115 Iasi, Romania; andreea.decusara8@gmail.com; 2 Institute of Gastroenterology and Hepatology, St. Spiridon Iasi County Emergency Clinical Hospital, Grigore T. Popa University of Medicine and Pharmacy, 700115 Iasi, Romania; toader.elena@yahoo.com; 3 Department of Anesthesia and Intensive Care, St. Spiridon Iasi County Emergency Clinical Hospital, Grigore T. Popa University of Medicine and Pharmacy, 700115 Iasi, Romania; miblaj@yahoo.com (M.B.); adi.ionut80@yahoo.com (A.-I.C.); 4 Department of Gastroenterology, School of Medicine, Carol Davila University of Medicine and Pharmacy, 050474 Bucharest, Romania; drsandruvasile@gmail.com

**Keywords:** severe acute pancreatitis, pancreatic fluid collections, fungal infection, *Candida* spp., antifungal therapy

## Abstract

**Background:** Fungal infection of pancreatic fluid collections (PFCs) in severe acute pancreatitis (SAP) is under-recognized and associated with poor outcomes. Overlap with bacterial infections and the need for invasive sampling often delay diagnosis, leading to prolonged antibiotic use without the use of antifungal agents. **Methods:** We report three cases of SAP complicated by fungal infection of PFCs. Two patients, one with alcohol-related pancreatitis and the other with biliary pancreatitis, developed symptomatic encapsulated necrosis. Both were successfully managed with endoscopic drainage and targeted antifungal therapy against *Candida albicans*, achieving full resolution. The third patient, with necrotizing biliary pancreatitis, underwent multiple surgical and endoscopic interventions and developed an infection with a *non-albicans Candida* species. Reduced susceptibility requires individualized antifungal adjustment guided by susceptibility testing. Despite aggressive multimodal therapy, the patient progressed to multiorgan failure and died subsequently. **Results:** These cases emphasize the clinical impact of fungal infections in patients with SAP, particularly their association with severe disease, prolonged hospitalization, and prior antibiotic exposure. These findings highlight the prognostic value of early microbiological sampling, species-level identification, and prompt initiation of antifungal therapy. Infections caused by non-*albicans* species pose additional challenges due to their reduced sensitivity to standard antifungal agents. **Conclusions:** Fungal infection of PFCs is a clinically significant and frequently underestimated complication of SAP. Early recognition and species-directed antifungal therapy are critical for improving outcomes in high-risk patients.

## 1. Introduction

Acute pancreatitis (AP) is a common gastrointestinal emergency with outcomes ranging from mild self-limiting forms to severe necrotizing disease. Approximately 20% of patients develop necrosis, a condition associated with significant morbidity and mortality, particularly when complicated by infection events [1].

In severe acute pancreatitis (SAP), sustained pancreatic injury accompanied by intense inflammation and parenchymal necrosis may lead to the development of local complications, such as pancreatic fluid collections (PFCs) [1,2].

The revised Atlanta classification (2012) identifies four types of PFCs based on their content and timing: acute peripancreatic fluid collections, acute necrotic collections, pseudocysts, and walled-off necrosis (WON) [2]. Although sterile at onset, pancreatic necrosis and PFCs may become infected in up to 40% of patients during the disease course [3]. These infectious complications pose a major therapeutic challenge, as septic events, typically arising after 10–14 days, remain the leading cause of late mortality in necrotizing pancreatitis [4].

Bacterial infection is widely recognized as a hallmark of severe pancreatitis, with an incidence of 20–40% [5]. However, fungal infections, most commonly due to *Candida* species, are less consistently reported, with highly variable incidence rates (6–46%), reflecting differences in study populations and diagnostic methods [6]. The true prevalence is likely underestimated because fungal cultures are not routinely performed, and undersampling remains common.

Fungal infection of pancreatic collections is strongly associated with worse outcomes and has been identified as an independent predictor of mortality, with reported death rates exceeding 50% in certain cohorts [7].

Despite their clinical and prognostic relevance, fungal infections of PFCs are frequently under-recognized. This is largely due to the overlap of clinical and imaging features with bacterial infection, combined with the fact that definitive microbiological confirmation requires invasive sampling [8]. As a result, diagnosis is frequently delayed, leading to prolonged antibiotic therapy without antifungal coverage and subsequent disease progression.

As early diagnosis and treatment can significantly improve outcomes, maintaining a high index of suspicion, particularly in patients with risk factors, is essential. This case series highlights the importance of early fungal screening in patients with PFCs, particularly those with persistent sepsis despite adequate antibacterial therapy. Cases were selected from approximately 250 new cases of moderate-to-severe acute pancreatitis admitted to our tertiary gastroenterology department within the last 12 months. Among these patients, only 23 required EUS-guided drainage for peripancreatic fluid collections and WONs. The three cases are the only locally reported fungal infections within the last five years. A comparison of these cases illustrates how the timing of diagnosis and initiation of antifungal therapy has a major impact on patient outcomes.

## 2. Methodology of Fungal Identification

### 2.1. Collection, Inoculation and Incubation

All samples were collected in sterile containers before any antifungal treatment was administered, following the protocols, and inoculated on Sabouraud media (Thermo Scientific™ Oxoid™ Sabouraud Dextrose Agar, Waltham, MA, USA) containing Chloramphenicol and Gentamicin. The procedure continued with an incubation period of 18 ± 2 h at 35 ± 2 °C, after which the plates were stored at room temperature for four additional days and assessed daily until reaching the endpoint of five days (120 h) post-incubation.

### 2.2. Identification

Fungal spectra were analyzed using Matrix-Assisted Laser Desorption Ionization Time-of-Flight Mass Spectrometry (MALDI Biotyper^®^ Sirius IVD System, version 12.0; Bruker Daltonics GmbH & Co. KG, Bremen, Germany).

### 2.3. Sensitivity Testing

Antifungal sensitivity testing was performed using the MICRONAUT-AM (Bruker, Billerica, MA, USA) automatic system. Employing the EUCAST methodology, the colorimetric broth microdilution-based MICRONAUT-AM (MCN-AM) assay is a commercial test for determining the minimal inhibitory concentration (MIC) of yeasts to antifungal drugs. To determine the MIC of our isolates, antifungal susceptibility testing was performed using the broth microdilution procedure of EUCAST v12.0.

## 3. Case Series

### 3.1. Case 1

**Patient profile:** A 27-year-old man with a history of recurrent alcohol-induced acute pancreatitis (three prior mild–moderate episodes managed conservatively). The last episode of mild acute pancreatitis occurred 6 months ago and involved no local complications. The patient fully recovered after conservative treatment. The full patient profiles are presented in Table 1.

**Clinical course:** He presented with a new (48 h since the onset of pain) severe episode of acute pancreatitis (modified CT Severity Index (mCTSI) at admission of 8 showing areas of acute necrosis and acute fluid collections), with fever, abdominal pain, and systemic inflammatory response (tachycardia, tachypnea, CRP 50 mg/dL, leukocytosis 20,000/mm^3^). Despite conservative treatment and empiric broad-spectrum antibiotics with 500 mg IV Imipenem–cilastatin every 8 h initiated because of baseline procalcitonin levels reaching 2.5 µg/L, his condition worsened 9 days after admission. Follow-up CT scan after 10 days of hospitalization revealed multiple peripancreatic necrotic collections and a symptomatic peripancreatic/subhepatic early organizing WON measuring 9 cm (with wall maturation ≥ 2 mm and 40% necrosis).

**Figure 1:**
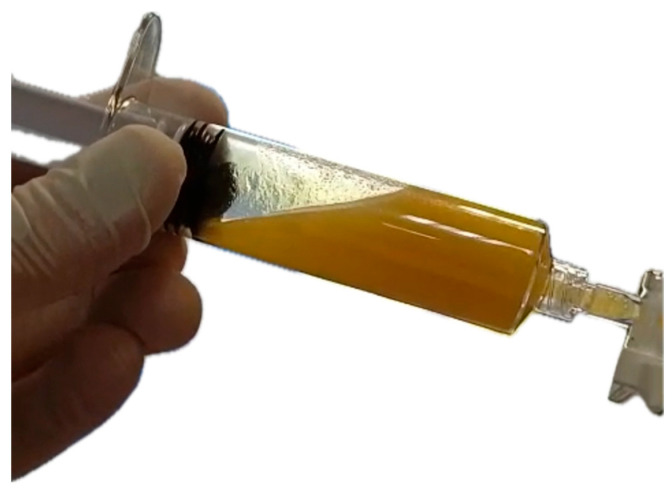
Macroscopic aspect of fluid during aspiration using FNA 19 Ga Needle (Boston Scientific, Marlborough, MA, USA).

**Intervention:** Endoscopic ultrasound (EUS)-guided fluid sampling by fine-needle aspiration (FNA) followed by cystogastrostomy with placement of a 15 × 10 mm lumen-apposing metal stent (LAMS) was performed on the 10th day of hospitalization, achieving effective drainage. Given the worsening condition despite broad-spectrum antibiotic therapy, FNA was performed before drainage to ensure proper fluid culture. The macroscopic appearance of the pseudocyst fluid during aspiration is shown in Figure 1, resembling a chylous aspirate.

**Microbiology and treatment:** Cultures isolated *Candida albicans*. Antifungal therapy was initiated with intravenous fluconazole 600 mg as a loading dose (10 mg/kg IV), followed by oral fluconazole 350 mg (≈6 mg/kg PO) daily for 14 days.

**Figure 2:**
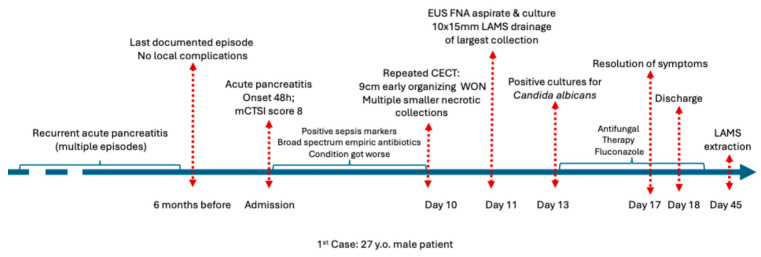
Chronological overview of the clinical course of Case 1 before admission, during hospitalization, and after discharge. Abbreviations: CECT (Contrast enhanced computed tomography).

**Outcome:** The patient improved rapidly 4 days after the initiation of antifungal therapy and was discharged within 24 h of an asymptomatic hospital stay. No necrosectomy was needed. Resolution of systemic inflammation and complete regression of early WON on follow-up imaging were confirmed within four weeks. The LAMS was removed at four weeks without recurrence. Figure 2 illustrates the complete chronological course of the patient.

### 3.2. Case 2

**Patient profile:** A 71-year-old woman with a history of well-controlled type 2 diabetes mellitus (HbA1c of 6.5%) and choledocholithiasis documented 6 months before the current admission was managed with ERCP and stone extraction. Surgical evaluation contraindicated cholecystectomy because of patient frailty due to sarcopenia and chronic coronary disease, which was non-amendable to angioplasty and stent insertion. Full patient profile can be found in Table 1.

**Figure 3:**
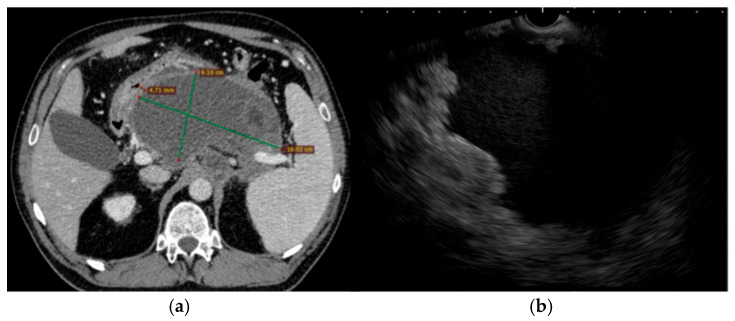
Pre-intervention imaging evaluation. (**a**). Abdominal CT scan showing a large peripancreatic retrogastric WON (with approximately 30% necrosis) with no parietal pseudoaneurysms, but with marked gastric and mesenteric compression; (**b**). Trans-gastric EUS image of the same collection of fluid.

**Clinical course:** The patient was admitted with acute cholangitis and spontaneous stone passage complicated by secondary biliary pancreatitis. The onset of symptoms has been reported to occur within under 24 h before admission. Presepsin at admission reached 1200 pg/mL, and procalcitonin was 0.75 µg/L. Despite supportive and antibiotic therapy with imipenem–cilastatin 500 mg IV every 8 h, she developed persistent fever, abdominal discomfort, and an ongoing inflammatory response. Contrast-enhanced CT scan within 72 h of admission reported an mCTSI score of 8 and revealed a large (9 × 16 cm) retrogastric peripancreatic collection with necrotic components and a mature wall consistent with a WON (Figure 3). No pseudoaneurysms were observed. Moderately dilated bile ducts have also been described.

**Figure 4:**
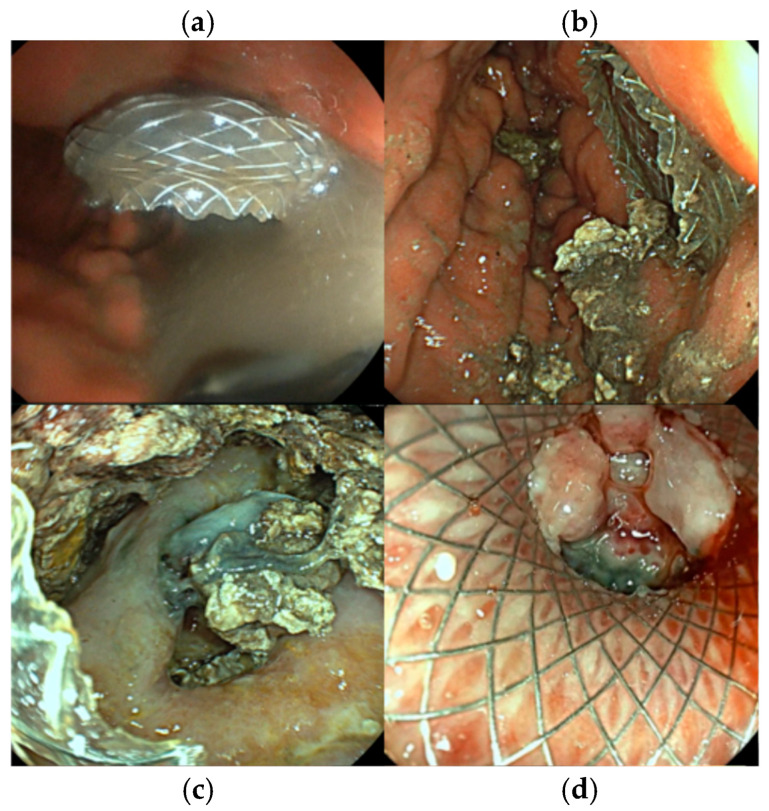
Endoscopic images of the same patient during multiple examinations. (**a**). aspect of fluid after stent deployment, (**b**). necrotic tissue during first necrosectomy, (**c**). detection of thrombosed vessels within the WON, (**d**). WON resolution showing a vital fibrous wall covering the LAMS opening, allowing LAMS extraction.

**Intervention:** EUS-guided cystogastrostomy with 15 × 10 mm LAMS placement was performed on the 5th day of hospitalization, allowing drainage of the collection and necrosectomy. Through-the-scope fluid aspiration was achieved using an ERCP cannula inserted into the cavity after stent deployment. Subsequently, the same procedure of stent dilation using a 12–15 mm CRE dilation balloon followed by necrosectomy was performed. Clinical remission of SIRS was achieved after the first necrosectomy session. The patient required another necrosectomy session after 24 h to achieve complete necrosis drainage. Thrombosed vessels were detected during necrosectomy; however, there was no indication for additional hemostatic procedures (Figure 4).

**Microbiology and treatment:** Cultures from the drained fluid grew *Candida albicans*. Antifungigram showed nystatin resistance and fluconazole sensitivity. Antifungal therapy with fluconazole 800 mg as a loading dose (10 mg/kg IV), followed by oral fluconazole 500 mg (≈6 mg/kg PO) daily for 14 days, was added to the ongoing antibiotics and supportive care.

**Figure 5:**
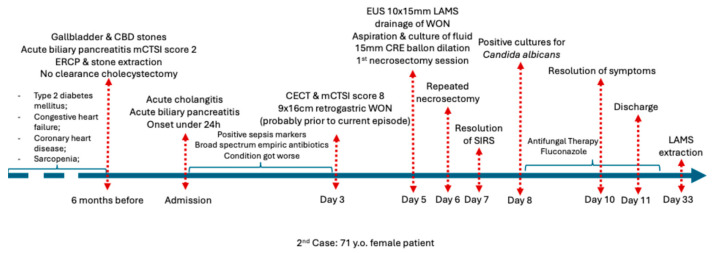
Chronological overview of the clinical course of Case 2 before admission, during hospitalization, and after discharge. Abbreviations: CECT (Contrast enhanced computed tomography), CRE (Controlled radial expansion).

**Outcome:** The patient showed gradual clinical and biochemical recovery, with resolution of SIRS 24 h after 2nd necrosectomy. Four days after the two necrosectomy sessions, the patient became asymptomatic and was discharged 24 h later. Complete regression of the pseudocyst was confirmed on routine follow-up imaging and endoscopic evaluation after one month, allowing stent removal. Figure 5 summarizes the chronological clinical course of the patient.

### 3.3. Case 3

**Patient profile:** A 54-year-old woman with a history of biliary pancreatitis and spontaneous choledochal stone passage (December 2023) based on magnetic resonance cholangiography follow-up showing gallbladder stones and a clear common bile duct. The patient did not present for follow-up evaluations and refused cholecystectomy. Full patient profile can be found in Table 1.

**Figure 6:**
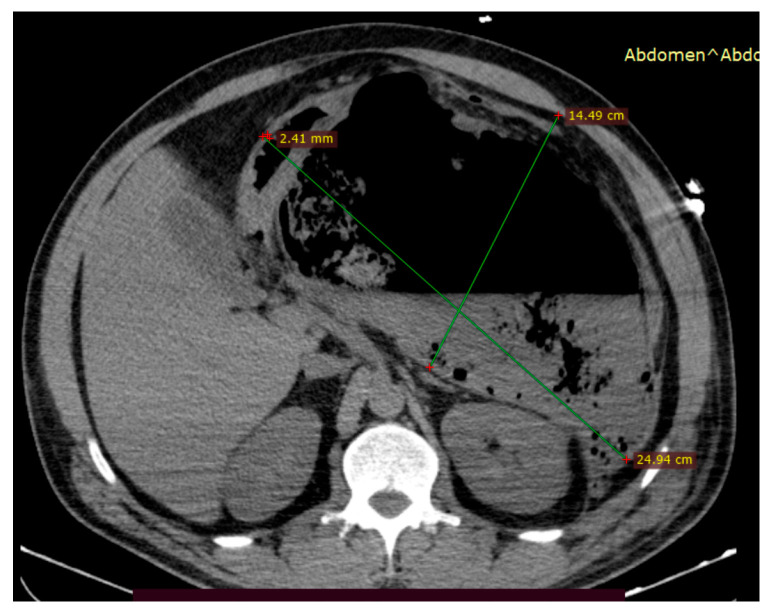
An abdominal CT scan performed within 24 h of admission showed a large mixed-type partially encapsulated peripancreatic necrotic collection (approximately 50% necrosis) causing organ compression and severe abdominal compartment syndrome.

**Clinical course:** She was readmitted one year after the last evaluation (30 December 2024) in critical condition with severe abdominal pain for more than 72 h prior to admission. The patient described multiple prior episodes of severe abdominal pain over the last month, which were managed with over-the-counter paracetamol. Contrast-enhanced CT scan performed in the emergency department showed severe necrotizing pancreatitis with an mCTSI score of 10, involving a 25 × 15 cm compressive and partially encapsulated peripancreatic collection causing compartment syndrome, as shown in Figure 6, along with bilateral pleural effusions. The intra-abdominal pressure 8 h after admission was 290 mmH_2_O. Exploratory laparotomy was performed due to the lack of a fully matured wall and revealed pancreatic necrosis with peritonitis; lavage and drainage of the necrotic tissue were performed.

**Postoperative course and reinterventions:** Re-laparotomy on 6 January 2025, was performed for lavage and abscess drainage. The patient developed persistent sepsis and was referred for endoscopic intervention to ensure optimized drainage. EUS evaluation was performed 6 days after the second laparotomy and showed an encapsulated retrogastric WON measuring 95/105 mm with a 3 mm wall and 80% necrosis, which was drained using a 10 × 15 mm LAMS insertion followed by necrosectomy. Intraprocedural cannula-guided fluid aspiration revealed multidrug-resistant *Candida glabrata* (*Nakaseomyces glabrata*). Subsequently, multiple endoscopic interventions, including lavage and necrosectomy, were performed.

**Microbiology and treatment:** Intraoperative and post-endoscopic aspiration cultures identified *Escherichia coli*, *Pseudomonas aeruginosa*, *Enterococcus faecium*, *Acinetobacter baumannii*, and *Staphylococcus aureus*. Fungal superinfection with *Candida glabrata* (*Nakaseomyces glabrata*) was confirmed only after EUS-guided drainage and aspiration. As stated by the local antibiotic use protocol, the patient received broad-spectrum antibiotics first with perioperative cefuroxime 1.5 g IV every 8 h for 48 h, imipenem–cilastatin 500 mg IV every 8 h for 14 days, and targeted antifungal therapy with caspofungin, as guided by susceptibility testing, with a loading dose of 70 mg IV followed by a continuous regimen of 50 mg IV each day throughout the hospital stay.

**Associated conditions** included inflammatory anemia, sepsis-related thrombocytopenia, and a grade II sacral pressure ulcer (grade II). Later, the patient developed pulmonary embolism, bilateral pneumonia secondary to long-term mechanical ventilation, hypokalemia, ascites, and progressive multiorgan failure.

**Figure 7:**
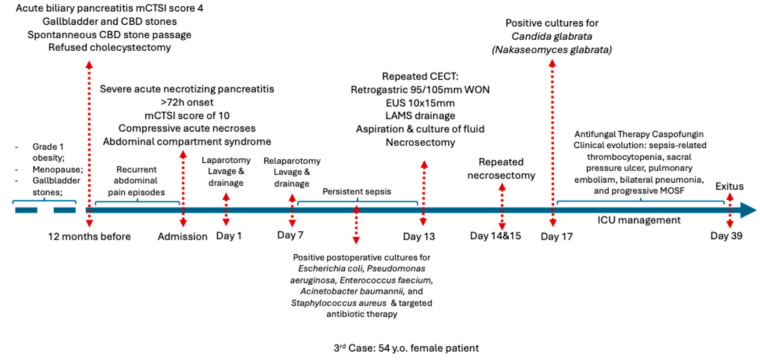
Chronological overview of the clinical course of Case 2 before admission, during hospitalization, and after discharge. Abbreviations: CECT (Contrast enhanced computed tomography), CBD (common bile duct), ICU (Intensive care unit), MOSF (Multiple System Organ Failure).

**Outcome:** Despite multimodal treatment (surgery, endoscopy, antimicrobials, and intensive care management), her condition deteriorated with recurrent resuscitated cardiac arrest, and she died after more than one month of intensive care hospitalization, as summarized in Figure 7.

## 4. Discussion

Fungal infections in SAP are increasingly recognized as key drivers of adverse effects. Unlike bacterial superinfection, fungal colonization of pancreatic necrosis remains less predictable, with a reported prevalence ranging from 7.6% to 46.3%, depending on patient characteristics and the diagnostic approach [9,10,11]. Such variability reflects heterogeneity in disease severity, comorbid burden, prior antibiotic exposure, and the extent of invasive procedures, as well as differences in diagnostic methods, from pancreatic tissue or blood cultures to β-D-glucan antigenemia testing [6]. In our series, fungal infection was confirmed in all three patients by positive cultures obtained directly from the peripancreatic collections.

The prevalence of fungal involvement increases sharply with disease progression, reaching 40–45% in patients with walled-off necrosis [9,11]. Importantly, this risk appears to be largely independent of pancreatitis etiology and instead reflects the severity of the disease and necrotic complications [12]. Our cases illustrate this principle: one patient with alcohol-related pancreatitis and two with biliary pancreatitis developed fungal infections, with the unifying feature being a severe and complicated clinical course rather than the underlying etiology.

Multiple predisposing factors for fungal infections in SAP have been described, including critical illness–associated immune dysfunction, prolonged broad-spectrum antibiotic use with intestinal fungal overgrowth, extended hospitalization, repeated surgical or endoscopic interventions, total parenteral nutrition, and mechanical ventilation [12]. Moreover, patients with type 2 diabetes mellitus face an elevated risk of major intra-abdominal fungal infections, primarily due to hyperglycemia-induced immune dysfunction, including impaired neutrophil function and reduced phagocytosis, which facilitate opportunistic pathogen translocation in peridigestive collections. In our series, prolonged hospitalization and antibiotic therapy in the setting of sepsis were present in all patients. The second patient was diabetic, although the condition was well controlled. In the third case, the cumulative burden of repeated surgical interventions further amplified the risk and was associated with the most unfavorable outcome. Nevertheless, due to impaired gut barrier function and translocation of gut fungi like Candida species directly into necrotic pancreatic tissues or fluids, independent of bloodstream dissemination. Thus, no patient developed fungemia.

From a pathophysiological perspective, the increased intestinal permeability observed in SAP facilitates the translocation of microorganisms from the gut lumen to extraintestinal sites, thereby contributing significantly to the pathogenesis of infection [13,14]. Mounting evidence indicates that gut-derived fungi can colonize necrotic pancreatic tissue [11,15]. Among these, *Candida* species, particularly *Candida albicans*, are the predominant commensals of the gastrointestinal mycobiome and are the most frequently isolated fungal organisms in patients with SAP. Less frequently, *C. glabrata, C. krusei,* and *C. tropicalis* have also been reported [12]. Their occurrence is explained by their role as commensal organisms in the gastrointestinal tract and their opportunistic pathogenic potential under conditions of immunosuppression and severe systemic inflammation. In a cohort of 136 patients with necrotizing pancreatitis, Rasch et al. (2018) isolated *Candida* spp. from pancreatic tissue in 54 cases and demonstrated significantly higher mortality in infected patients than in non-infected patients (35.2% vs. 13.4%) [11]. Similarly, Werge et al. (2016) reported fungal isolation in nearly half of patients with walled-off necrosis (46%), supporting the hypothesis that fungal colonization may influence the maturation and encapsulation of necrosis, a concept further reinforced by Otsuka et al. (2024) [9,16].

In our case series, *Candida albicans* was identified in the first two patients, both of whom had favorable outcomes with targeted therapy. In contrast, in the third case, which had an unfavorable clinical course, another *Candida* species, *Candida glabrata*, was isolated. According to the literature, *C. glabrata* possesses distinct pathogenic characteristics and reduced susceptibility to commonly used antifungal agents (azoles and echinocandins), which may contribute to its association with worse outcomes [17].

Our cases confirm that fungal infection in PFCs is not merely an incidental finding but a clinically significant complication. All three patients developed symptomatic collections and a persistent inflammatory response that prompted drainage and microbiological sampling of the abscess. In each case, fungal pathogens were identified, and antifungal therapy was administered. This highlights two important aspects: (1) fungal infections may be underdiagnosed in routine practice due to a lack of targeted sampling, and (2) drainage procedures serve a dual role—therapeutic decompression and etiological diagnosis through culture.

Another important observation is the impact on prognosis. Several studies have identified fungal infections as independent predictors of mortality in SAP, with reported death rates exceeding 50% in some cohorts [7,18]. In our series, death was recorded in one of the three cases, namely the patient who required repeated surgical interventions, with cultures confirming a polymicrobial infection involving *Escherichia coli* and *Pseudomonas aeruginosa*, which was subsequently complicated by the isolation of *Candida glabrata*. This species is known for its reduced susceptibility to commonly used antifungal agents, which makes the establishment of prompt therapy particularly challenging and contributes to treatment delays. These findings underscore the importance of accurate species identification and timely initiation of antifungal therapy as critical determinants of prognosis.

The selection of an antifungal agent requires careful evaluation. Azoles, which penetrate pancreatic tissue well, are widely used as first-line therapy and are highly effective against *Candida albicans* [19]. In contrast, resistance to azoles has been increasingly reported among non-*albicans Candida* species, especially *Candida glabrata*, which was isolated from the third patient. In such situations, echinocandins are the recommended first-line option; however, the emergence of multidrug-resistant strains highlights the need for routine antifungal susceptibility testing [19,20]. Therefore, species-level identification is essential because susceptibility patterns vary. In our series, clinical improvement was achieved with azole therapy (fluconazole) in patients with *Candida albicans* infection, both of whom experienced favorable outcomes. In contrast, in the case of an unfavorable outcome, therapeutic management was more complex, requiring antifungal adjustment according to susceptibility testing and the introduction of an echinocandin (caspofungin), consistent with reports emphasizing the importance of species-guided therapy [7,21].

The role of prophylactic antifungal therapy in SAP remains controversial. Some studies have suggested that early fluconazole may reduce infection rates [22,23], but meta-analyses and guidelines consistently advise against prophylaxis, as survival benefit has not been demonstrated, and prophylaxis may even increase the risk of resistant infections [24,25,26]. Our cases support the notion that empirical antifungal coverage should not be routine but should be considered in patients with high-risk features—prolonged antibiotic use, persistent sepsis, multiple interventions, or organ failure—while awaiting microbiological confirmation.

From a broader perspective, the link between fungi and SAP raises intriguing mechanistic concerns. Experimental models have demonstrated that fungi can amplify pro-inflammatory cytokine cascades, potentially aggravating pancreatic and intestinal barrier injury [14]. Future research using next-generation sequencing of pancreatic and blood samples could help define the fungal community more comprehensively beyond conventional cultures and clarify the role of fungal dysbiosis in SAP [27].

This case series has several important limitations that must be acknowledged when interpreting its findings in the context of existing literature. First, the very small sample size (three cases identified among approximately 250 patients with moderate-to-severe acute pancreatitis and 23 who underwent EUS-guided drainage) and the single-center, tertiary-referral setting substantially limit external validity and preclude any estimation of true incidence or robust risk stratification, in contrast to the larger cohorts by Werge et al. and Rasch et al., which included over 100 patients with necrotizing pancreatitis or walled-off necrosis and were able to more convincingly demonstrate the impact of fungal infection on morbidity and mortality [9,11,18]. Second, fungal infection was only investigated in patients undergoing invasive procedures, without a standardized institutional protocol for routine fungal screening (e.g., systematic sampling of all collections, serial cultures, or adjunctive β-D-glucan testing), which likely leads to underdiagnosis of early or subclinical fungal involvement and contrasts with studies that employed broader mycological workups or prospective sampling strategies [6,11,13,27]. Moreover, the choice of antifungal agent and duration (Fluconazole for *C. albicans* and Caspofungin for *C. glabrata*) were individualized according to local protocol recommendations, preventing comparison of different therapeutic strategies and limiting alignment with guideline-based algorithms that increasingly emphasize species-specific resistance patterns and standardized echinocandin use in non-albicans Candida infections [12,17,19,20,21].

Taken together, these limitations indicate that our findings should be regarded as hypothesis-generating and complementary to the existing evidence, underscoring real-world diagnostic and therapeutic challenges rather than providing definitive estimates of risk or treatment effects.

In summary, fungal infections in pancreatic collections are clinically significant and often under-recognized complications of SAP. Our case series reinforces the importance of early microbiological sampling of collections, particularly in patients with persistent sepsis despite antibiotics, and demonstrates that timely antifungal therapy can decisively influence outcomes. Larger prospective studies are needed to define the epidemiology, refine diagnostic strategies, and determine the optimal timing and choice of antifungal therapy for this high-risk patient group.

## 5. Conclusions

Fungal infections in SAP remain an underrecognized, clinically significant complication strongly associated with necrotic collections and adverse outcomes. Our case series illustrates how timely suspicion, early sampling, and targeted antifungal therapy can alter the disease trajectory, underscoring the importance of incorporating fungal screening into the management of high-risk patients.

## Figures and Tables

**Table 1:** Summary of Clinical and Biological Features of the Patients.

Category/Parameter	Case 1	Case 2	Case 3
Patient profile	27-year-old male, alcohol-related pancreatitis	71-year-old female, biliary pancreatitis, cardiovascular and metabolic comorbidities	54-year-old female, biliary pancreatitis
Clinical presentation	Fever, abdominal pain, tachycardia, tachypnea; SIRS	Fever, abdominal pain; tachycardia, SIRS	Critical condition; severe abdominal pain; compartment syndrome; severe sepsis
Blood pressure	126/76 mmHg	148/89 mmHg	72/53 mmHg
Heart rate	116 bpm	101 bpm	126 bpm
Respiratory status	No respiratory failure	No respiratory failure	Required prolonged mechanical ventilation > 96 h; bilateral pneumonia
Temperature	Fever	Fever	fever/hypothermia fluctuations
ICU stay	No	No	>1 month ICU
Need for vasopressors	No	No	norepinephrine
Intra-abdominal pressure	Not reported	Not reported	290 mmH_2_O (abdominal compartment syndrome)
CRP (mg/dL, 0–0.5)	50	27	20
Procalcitonin (µg/L, <0.05)	2.5 µg/L	Not reported	Not reported
Leukocytes (×10^3^/mm^3^, normal 4–10)	20,000/mm^3^	11,000/mm^3^	47,000/mm^3^
Hemoglobin (g/dL, normal 12–16)	18.2	9.3	6.3
Platelets (×10^3^/mm^3^, normal 150–400)	449	587	96
Coagulation PT (s, 11–13.5) INR (0.8–1.2)	11 1.3	13 1.2	18 1.8
Lipase (U/L, <60)	1981	1174	>12,000
Amylase (U/L, <110)	471	598	5392
Liver function tests (at admittance) AST (U/L, <40) ALT (U/L, <40) GGT (U/L, <40) ALP (U/L, 40–130) BT (mg/dL, <1.2) BD (mg/dL, <0.3)	191 273 156 471 1.29 0.55	688 157 1227 442 9.7 6.95	601 575 253 663 1.86 0.92
Electrolytes Na^+^ (mmol/L; 135–145) K^+^ (mmol/L; 3.5–5.1)	134 3.7	132 3.6	126 2.7
Glucose (mg/dL, 70–99)	196	225	213
Creatinine (mg/dL, 0.6–1.3)	0.7	1.2	2.1
Major risk factors for fungal infection	Prolonged antibiotics > 10 days; mature and large WON;	Type 2 diabetes melitus Prolonged antibiotics; prolonged hospitalization; large WON; >1 endoscopic intervention	Long-term antibiotics; >1 month ICU; multiple collections; mechanical ventilation; TPN; repeated surgery; multiorgan failure
Imaging findings	Multiple necrotic peripancreatic collections; 9 cm WON	Large 9 × 16 cm retro gastric WON	Large 25 × 15 cm mixed-type collection with peritonitis Subsequently 95/105 mm retrogastric WON
Major interventions	EUS-FNA + LAMS	EUS-LAMS + 2 necrosectomies	Two laparotomies + EUS-LAMS + multiple necrosectomies
Organisms isolated (fungal)	*Candida albicans*	*Candida albicans* (nystatin-R, fluconazole-S)	*Candida glabrata*
Organisms isolated (bacterial)	None reported	None reported	*E. coli*, *Pseudomonas*, *Enterococcus faecium*, *Acinetobacter baumannii*, *S. aureus*
Antifungal therapy	Fluconazole 14 days	Fluconazole 14 days	Caspofungin 20 days
Associated complications	SIRS	Thrombosed vessels inside WON, SIRS	Anemia, thrombocytopenia, pulmonary embolism, pneumonia, ascites, multiorgan failure
Outcome	Full recovery; resolution of WON	Favorable recovery after drainage + necrosectomy	Progressive deterioration → multiorgan failure → death

Abbreviations: ALP (Alkaline phosphatase); ALT (Alanine aminotransferase); AST (Aspartate aminotransferase); BD (Direct bilirubin); BT (Total bilirubin); CRP (C-reactive protein); EUS (Endoscopic ultrasound); FNA (Fine-needle aspiration); GGT (Gamma-glutamyl transferase); ICU (Intensive care unit); INR (International normalized ratio); LAMS (Lumen-apposing metal stent); Na^+^ (Sodium); K^+^ (Potassium); PFCs (Pancreatic fluid collections); PT (Prothrombin time); SAP (Severe acute pancreatitis); SIRS (Systemic inflammatory response syndrome); TPN (Total parenteral nutrition); WON (Walled-off necrosis).

## Data Availability

Supporting data is available upon request from the corresponding author.
